# E2F1 Regulates Cellular Growth by mTORC1 Signaling

**DOI:** 10.1371/journal.pone.0016163

**Published:** 2011-01-24

**Authors:** Sebastian Real, Nathalie Meo-Evoli, Lilia Espada, Albert Tauler

**Affiliations:** Departament de Bioquímica i Biologia Molecular, Facultat de Farmàcia, Universitat de Barcelona, Barcelona, Spain; University of Birmingham, United Kingdom

## Abstract

During cell proliferation, growth must occur to maintain homeostatic cell size. Here we show that E2F1 is capable of inducing growth by regulating mTORC1 activity. The activation of cell growth and mTORC1 by E2F1 is dependent on both E2F1's ability to bind DNA and to regulate gene transcription, demonstrating that a gene induction expression program is required in this process. Unlike E2F1, E2F3 is unable to activate mTORC1, suggesting that growth activity could be restricted to individual E2F members. The effect of E2F1 on the activation of mTORC1 does not depend on Akt. Furthermore, over-expression of TSC2 does not interfere with the effect of E2F1, indicating that the E2F1-induced signal pathway can compensate for the inhibitory effect of TSC2 on Rheb. Immunolocalization studies demonstrate that E2F1 induces the translocation of mTORC1 to the late endosome vesicles, in a mechanism dependent of leucine. E2F1 and leucine, or insulin, together affect the activation of S6K stronger than alone suggesting that they are complementary in activating the signal pathway. From these studies, E2F1 emerges as a key protein that integrates cell division and growth, both of which are essential for cell proliferation.

## Introduction

For many years, the E2F family of transcription factors have been well known for their ability to regulate cell cycle progression by coordinating a large group of genes involved in G1 to S phase transition. However, numerous studies have shown that E2F activity could also promote the expression of genes that control cell death, differentiation and development programs [1]. In mammals, the E2F family is composed by eight members and the diversity found in this family reflects distinct roles in the transcriptional regulation and cell function. E2F1-3, forming heterodimers with DP proteins, function primarily as transcriptional activators; in contrast, E2F4-8 act mainly as transcriptional repressors. E2F transcriptional activity is mainly regulated by the retinoblastoma protein family. Among the eight members of the E2F family described, E2F1 is unique in its ability to induce apoptosis [2].

Although E2F is a key regulator of cell proliferation, its capacity to control cell growth is uncertain. During proliferation, increase of mass must occur to maintain homeostatic cell size during each cell cycle. Growth and cell division are coupled during the cell cycle; however, both processes are independently regulated. The question of whether E2F play a role in cell size in mitotic cells has been studied in depth, mainly in *Drosophila*. Over-expression of dE2F accelerates the cell cycle without affecting growth, and leads to a greater number of smaller cells [3]. From these results and others, investigators have discarded the idea of any hypothetic growth function of E2F. Interestingly, in this organism, cellular growth is controlled by Cyclin D-Cdk4 complex, an upstream regulator of E2F through the pRb pathway [4]. In mammalian cells, the role of E2F on growth has been less extensively studied and is still questionable. Over-expression of E2F1 in the liver does not produce any increase in hepatocytes' mass [5]. However, in contrast to this lack of growth function, E2F1 is found to be essential for the induction of hypertrophy in C2C12 myoblasts, a growth process that occurs without cell division [6].

Cellular growth requires modulation of protein synthesis. One of the rate-limiting steps in this process is the initiation of translation, a phase in which the mammalian target of the rapamycin complex1 (mTORC1) signaling pathway plays an essential role [7]. Activation of the kinase activity of mTORC1 drives phosphorylation and inactivation of the eukaryotic translation initiation factor 4E-binding protein 4E-BP, a repressor of translation initiation, and phosphorylation and activation of ribosomal protein S6 kinase 1 (S6K1) [8], [9]. mTORC1 is regulated by mitogens through a canonical signaling cascade triggered by the activation of class I PI3K and Akt [10]. Tuberous sclerosis genes, hamartin (TSC1) and tuberin (TSC2) are involved in this pathway. Acting as a complex, TSC1 and TSC2 negatively regulate mTOR1 by inactivating the small G protein Rheb through TSC2's GTPase activating protein activity. TSC1 is necessary for the stabilization of TSC2, preventing its ubiquitin-mediated degradation. Activation of Akt produces the phosphorylation and inhibition of the TSC1/TSC2 complex [11]. In addition to Akt, other kinases are capable of phosphorylating TSC2 and regulating its function in mTORC1 signaling, and these include: Erk2 and RSK, which inhibit the TSC2 activity, while AMPK and GSK3β activate it [12], [13].

In this study, we demonstrate for the first time that, in addition to its role in proliferation and apoptosis, E2F1 is capable of regulating cellular growth. E2F1 emerges as a key protein that integrates cell division and growth, essential processes for cell proliferation.

## Results

### E2F1 activity induces growth

**Figure 1:**
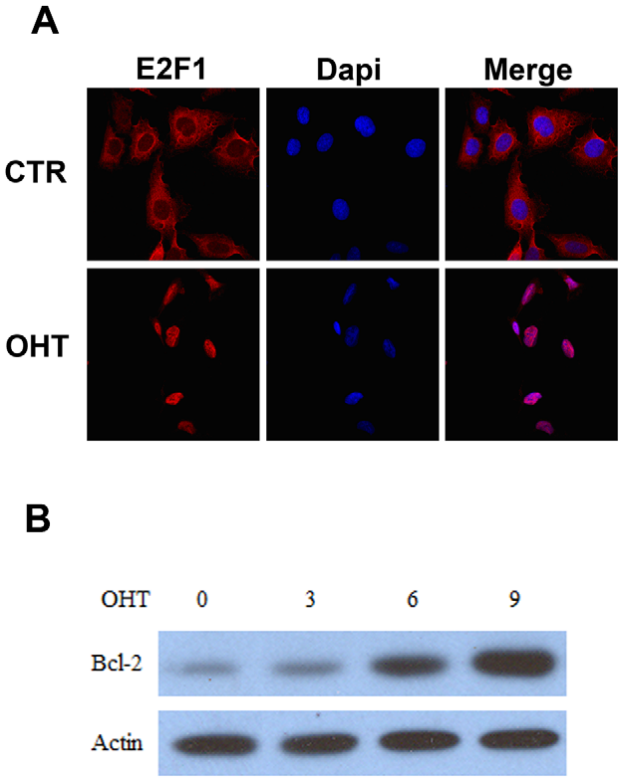
Effect of OHT on the translocation of ER-E2F1 to the nucleus and Bcl-2 expression. (A) Stable ER-E2F1 U2OS cells were serum-starved (control) and treated with OHT for 6 hours (OHT). Cells were immunostained for E2F1 protein (red) and DAPI (blue). (B) Stable ER-E2F1 U2OS cells were serum-starved and treated with OHT at the indicated times. Expression of the indicated proteins was determined by Western blot analysis.

**Figure 2:**
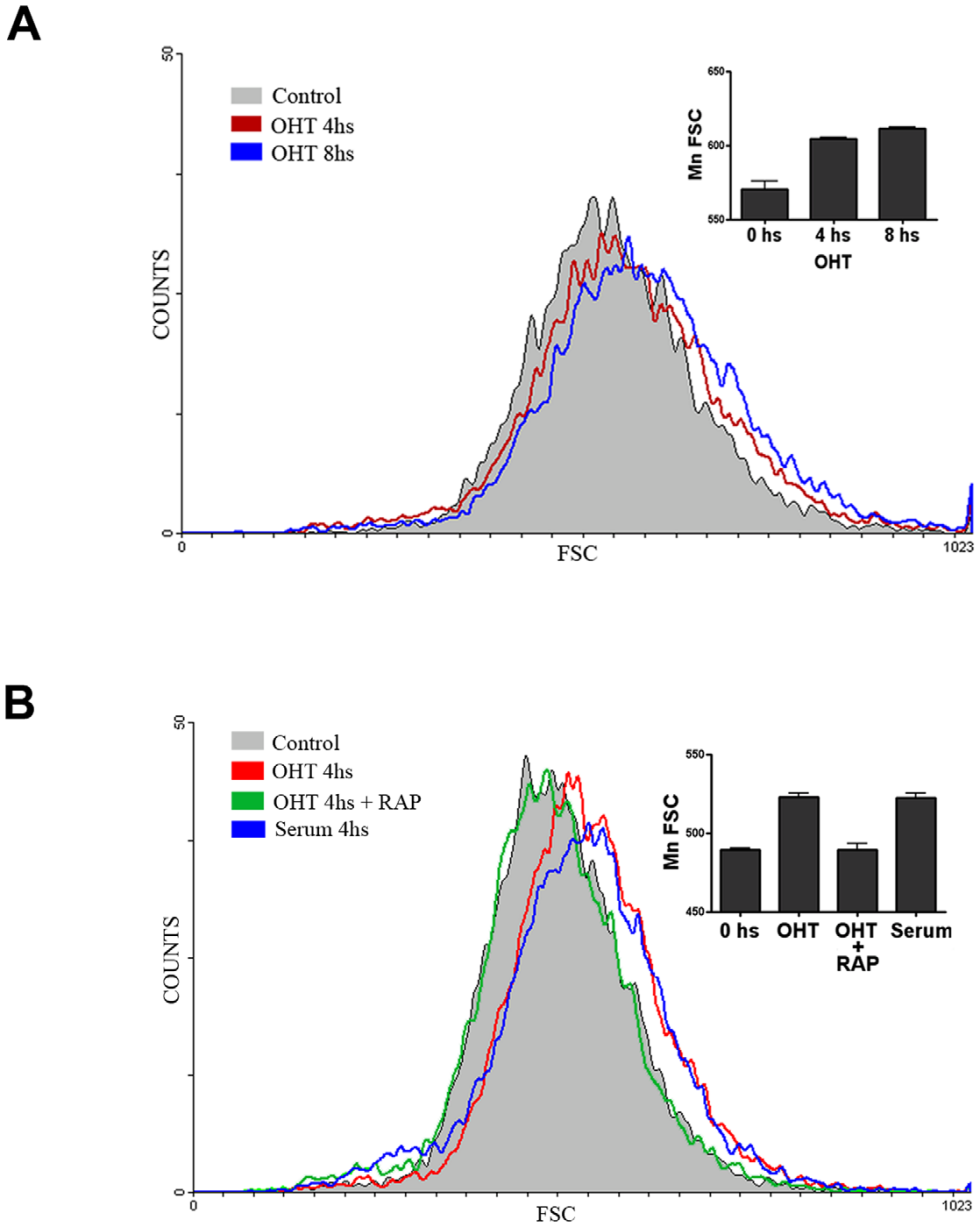
E2F1 control cellular size. (A) ER-E2F1 U2OS cells were serum-starved and treated with OHT at the indicated hours. Size distribution of G1 phase cells was analyzed by forward light scatter (FSC). (B) Size distribution of G1 phase cells was analyzed after treatment with serum, and OHT in the presence and absence of rapamycin (rap). Means of forward scatter populations are presented by graph in the left panels.

In order to study the growth effect of E2F1, we developed a U2OS ER-E2F1 stable cell line in which E2F1 activity is dependent on the presence of 4-hydroxitamoxifen (OHT) [14]. This system has been extensively used in other E2F1 function studies [15], [16]. The ER-E2F1 fusion protein is expressed in the cytosol and translocates to the nucleus in the presence of OHT; expression of the E2F1-regulated gene Bcl-2 was detected to confirm E2F1 action (Fig. 1A and 1B). We analyzed the relative cell size by flow cytometry using forward angle light scatter. Results demonstrated that activation of E2F1 by OHT addition promotes cell size increase in G1 cells in a time-dependent manner (Fig. 2A). The change in size observed was similar to that detected with serum addition (Fig. 2B). Prior studies have demonstrated the involvement of mTORC1 in the growth cell response to numerous stimuli [17]. To test whether this kinase participates in the increase in cell size detected after E2F1 induction, we analyzed the effect of the well-known mTORC1 inhibitor, rapamycin, on this response. Results obtained show that the addition of rapamycin completely blocked the growth effect of E2F1, suggesting that mTORC1 plays an essential role in this process (Fig. 2B). Addition of rapamycin did not interfere with the translocation of ER-E2F1 to the nuclei (Fig. S1). The increase on E2F1 expression found after OHT addition can be due to the effect on E2F1 protein stabilization that occurs in this cellular compartment [18].

### E2F1, but not E2F3 regulates mTOR1C by a transcriptional mechanism

**Figure 3:**
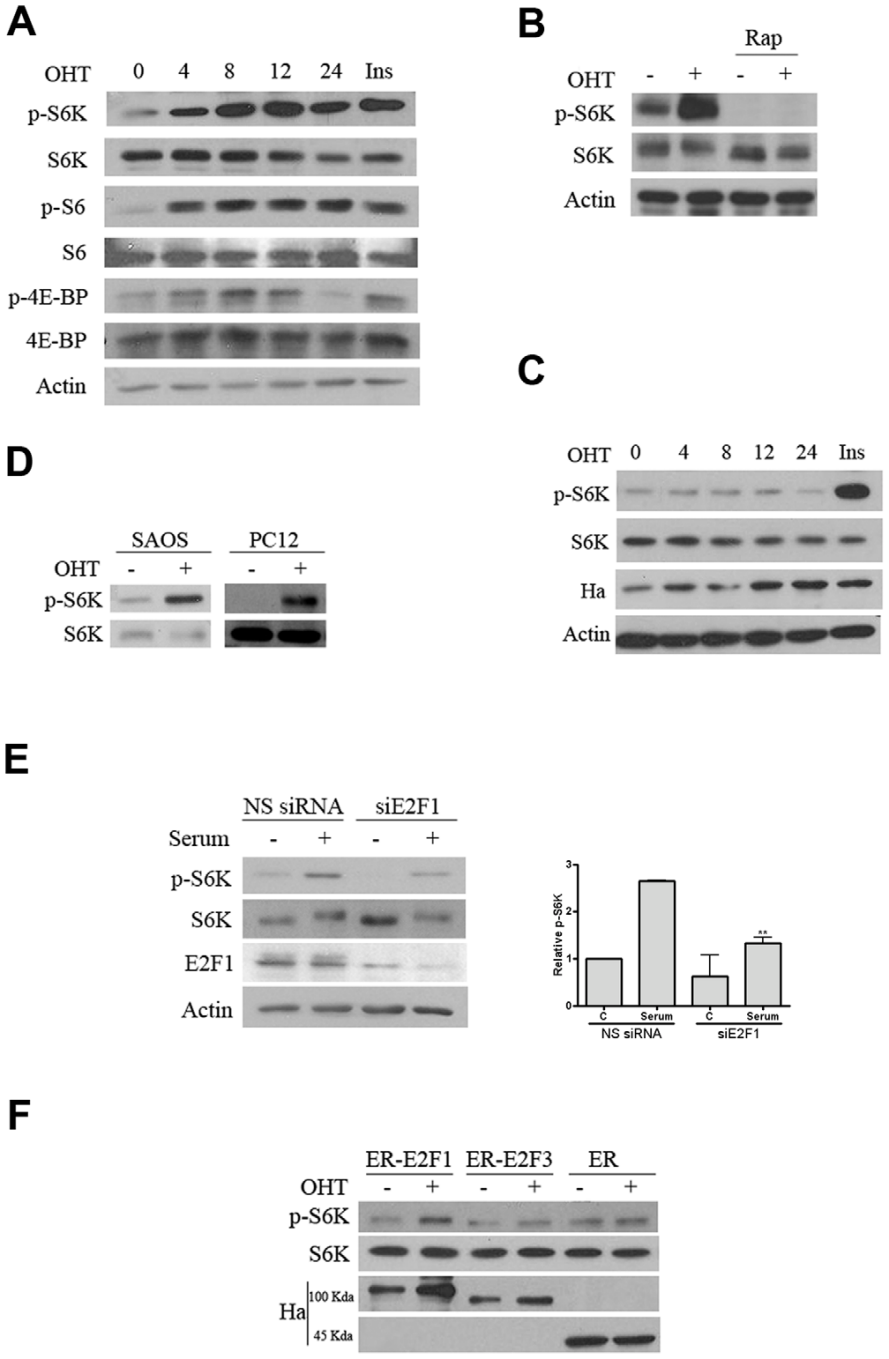
Effect of E2F1 on mTOR activity. Expression of the indicated proteins was determined by Western blot analysis. (A) Stable transfected ER-E2F1U2OS cells were serum-starved and treated with OHT at the indicated hours and with insulin (Ins) for 2 h or with OHT at the indicated hours. (B) Stable transfected ER-E2F1U2OS cells were serum-starved and treated with OHT (+) or not (−) for 6 h in the presence or absence of rapamycin (rap). (C) Stable transfected ER U2OS cells were serum-starved and treated with OHT at the indicated hours and with insulin (Ins) for 2 h or with OHT at the indicated hours. (D) Stable transfected ER-E2F1 SAOS and PC12 cells were serum-starved and treated with OHT (+) or not (−) for 6 h. (E) U2OS cells were transfected with E2F1 siRNA or non-silencing siRNA, serum-starved and treated with serum (+) or not (−) for 6 h. Quantification of Western blot from three independent experiments is shown on the right panel. (F) U2OS were transient transfected with the indicated expression vectors. After serum-starved, cells were treated with OHT (+) or not (−) for 6 h.

In vivo mTORC1 activity was analyzed by measuring the levels of the phosphorylated form of S6K and 4E-BP. Activation of E2F1 induced the phosphorylation of S6K and 4E-BP at Thr 398 and Thr 37/46 respectively, which started at 4 h and increased until 12 h following the treatment. Moreover, the increase in S6K phosphorylation correlated with an increase in S6K activity measured by phosphorylation of its own target, S6 (Fig. 3A). As expected, the addition of rapamycin totally abolished S6K phosphorylation (Fig. 3B). Note that ER alone did not produce phosphorylation of S6K, excluding the possibility of an artifact contribution of the ER sequence included in ER-E2F1 fusion protein (Fig. 3C). The ability of E2F1 to induce the activation of S6K was also studied in Saos-2 and PC12 cells, demonstrating that activation of mTORC1 by E2F1 is not a cell type phenomenon (Fig. 3D). Depletion of E2F1 by siRNA reduced the effect of E2F1 on S6K phosphorylation, which supports its role on the growth process (Fig. 3E).

**Figure 4:**
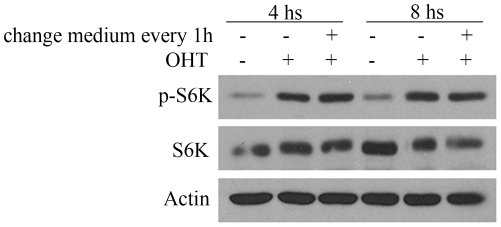
Effect of replacing the medium on S6K phosphorylation by E2F1. Stable transfected ER-E2F1U2OS cells were serum-starved and treated with OHT (+) or not (−) at the indicated times. Replace of medium to the cells were achieved every hour in the indicate conditions (+) or not (−). Expression of the indicated proteins was determined by Western blot analysis.

We further investigate whether the growth function of E2F1 could be extended to other members of the E2F family [19]. This possibility was tested by studying the change in mTORC1 activity in response to E2F3, an E2F family member that possesses exclusively a proliferative function. In contrast to E2F1, E2F3 was not able to induce the phosphorylation of S6K, suggesting that E2F growth activity is restricted to individual E2F members (Fig. 3F). To confirm that E2F3 was functional, we measured E2F3 transcriptional activity. To this end, we transiently transfected cells with ER-E2F3 and with an E2F-reported constructs, and luciferase activity was measured after OHT addition. The results obtained confirm the functionality of the E2F3 expression vector used (Fig. S2). Experiments in which the medium was removed and replaced multiple times during E2F1 activation demonstrated that activation of S6K by E2F1 was independent of any autocrine/ paracrine growth factors (Fig. 4).

**Figure 5:**
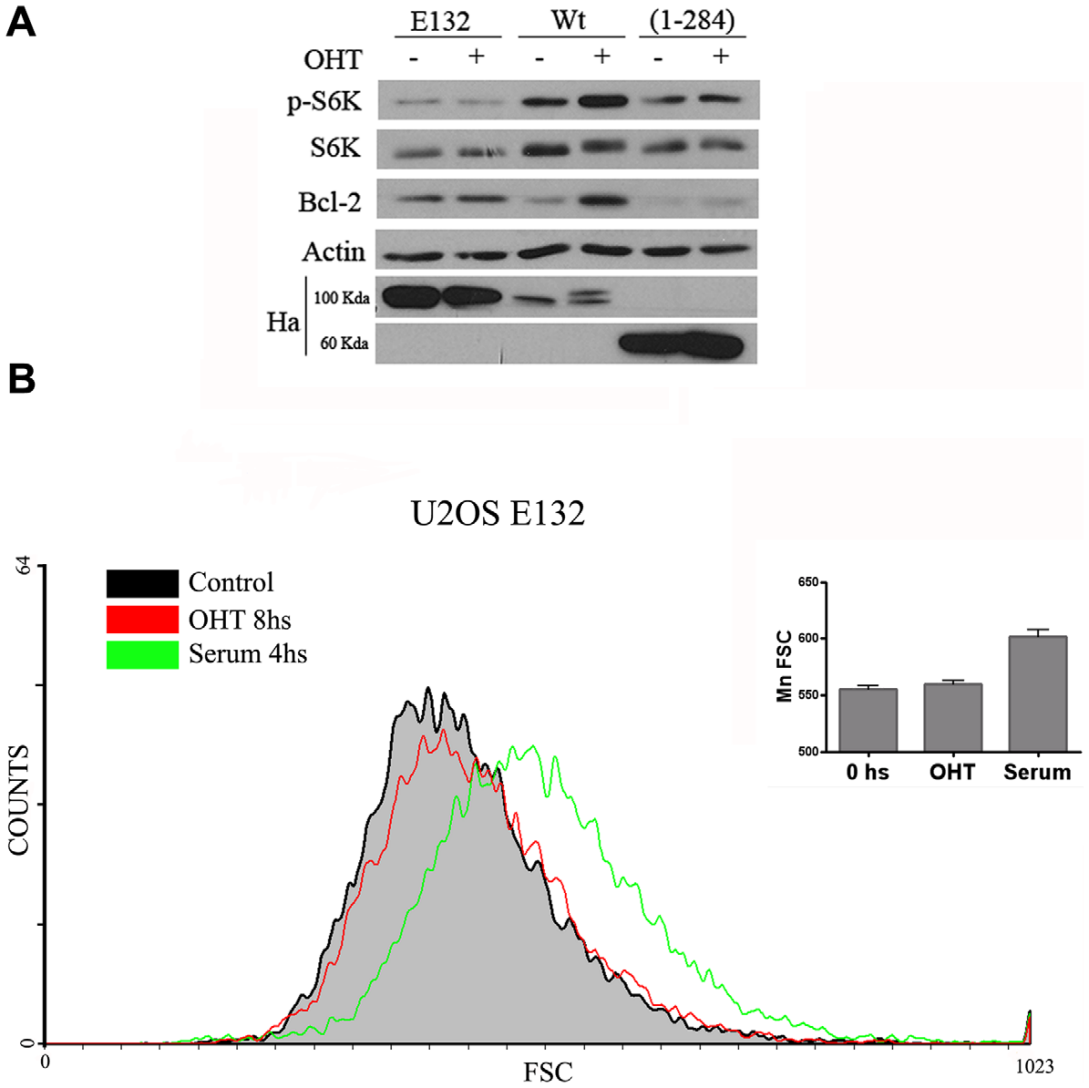
Effect of E2F1 mutants on mTOR activity and cellular size. (A) Stable transfected ER-E2F1 U2OS cells wild type (Wt) and mutants (E132, 1–284) were serum-starved and treated with OHT (+) or not (−) for 6 h. Expression of the indicated proteins was determined by Western blot analysis. (B) Stable transfected ER-E132 mutant (E132) was serum-starved and treated with OHT and serum as indicated. Size distribution of G1 phase cells was analyzed by forward light scatter (FSC). Means of forward scatter populations are presented by graph in the left panels.

Interestingly, E2F1 is also the only member of the E2F family of transcription factors that has a dual function in regulating either proliferation or apoptosis [20]. Numerous studies have established that the role of E2F1 in both processes could be a consequence of its specific capability to transcriptionally modulate essential genes for both processes. However, studies have shown that a mutant that lacks the transcriptional activation domain retains the ability to induce apoptosis, suggesting that E2F1 could also act through a non-transcriptional mechanism [21]. In order to determine whether the transactivation domain of E2F1 is required for its growth function, we studied the effect of an E2F1 mutant (E2F1_1–284_) lacking the transactivation binding domain of E2F1 on S6K phosphorylation. The results obtained demonstrate the essential role of this domain in the growth function of E2F1 (Fig. 5A). To confirm that E2F1 operates by a transcriptional mechanism, an E2F1 mutant (E2F1_E132_) lacking the DNA binding domain was tested for its ability to induce the phosphorylation of S6K. Over-expression of E2F1_E132_ does not promote the phosphorylation of S6K; thereby confirming that the E2F1 DNA binding domain is required for its growth function (Fig. 5A). Indeed, analysis of the relative cell size of cell lines stablely transfected with the E2F1_1–284_ mutant demonstrates that, as expected, OHT addition does not produce any change on the cell growth (Fig. 5B). Note that E2F1 mutants are expressed at a higher concentration than the wild type. Expression of Bcl2 was used as a positive control of the E2F1 regulated gene. Addition of OHT only increased the expression of Bcl-2 on cells expressing the wild type E2F1 but not in E2F1_E132_ and E2F1_1–284_ mutants, confirming that mutants are not able to activate transcription. Interestingly, in untreated cells, the Bcl-2 expression levels were different depending on which E2F1 construct cells had been transfected. This could be due to a non-transcription effect of E2F1, as has been reported for other E2F target genes [21]. Works are in progress to study the molecular basic of this effect.

### Activation of mTORC1 by E2F1 is independent of Akt and is not affected by over-expression of TSC2

**Figure 6:**
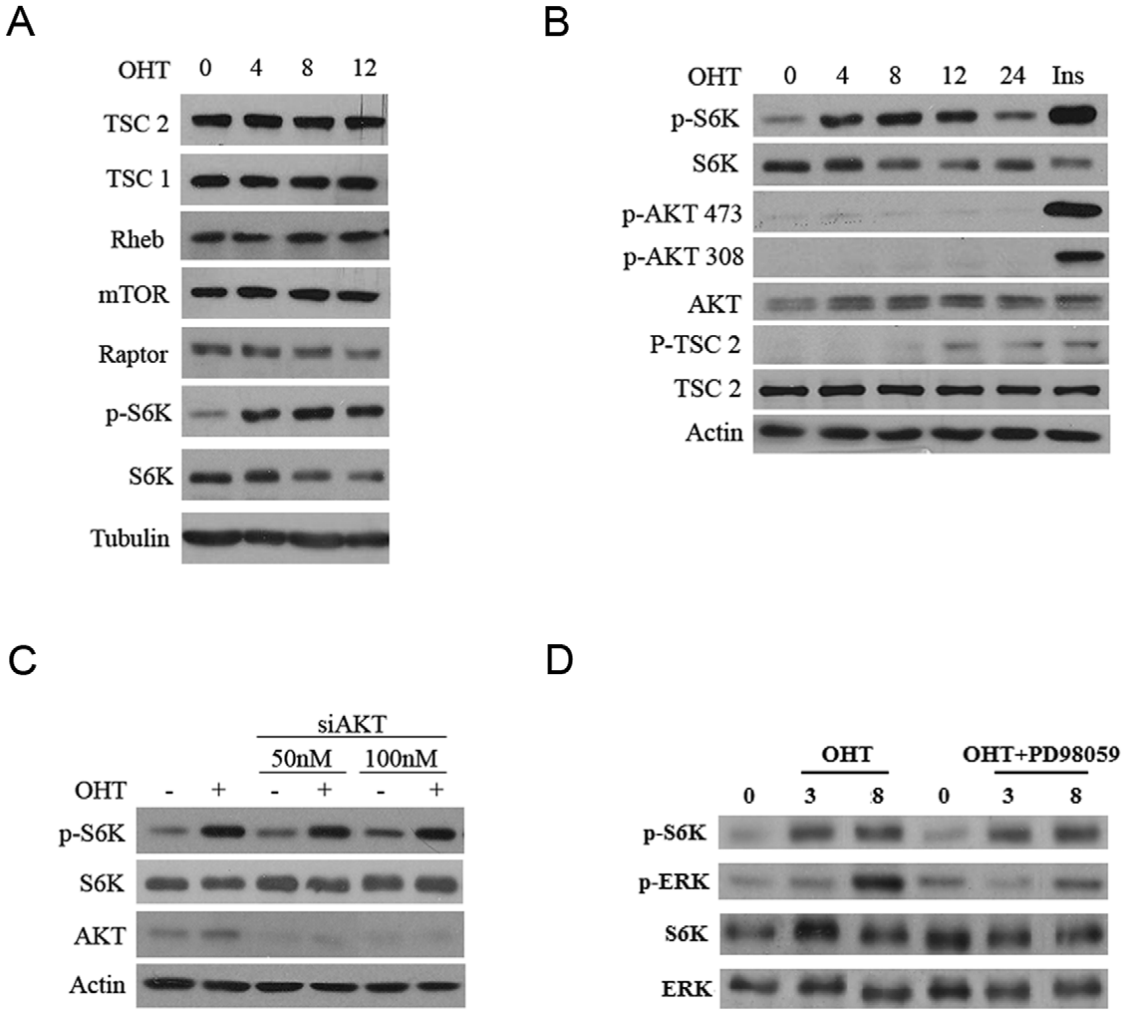
Effect of E2F1 on the TSC/mTOR signal transduction pathway. Expression of the indicated proteins was determined by Western blot analysis. (A, B) Stable ER-E2F1 U2OS cells were serum-starved and treated with OHT at the indicated hours. (C) Stable ER-E2F1 U2OS cells were transfected or not with Akt1 siRNA at the indicated concentrations, serum-starved and treated with OHT (+) or not (−) for 6 h. (D) Stable transfected ER-E2F1U2OS cells were serum-starved and treated with OHT at the indicated times in the presence or absence of PD98059.

In order to understand the mechanism by which E2F1 activates mTOR, we focused the study in the effect of E2F1 on the TSC/mTORC1 signaling pathway, the canonical pathway used for the regulation of growth in the proliferative processes. The obtained results demonstrate that E2F1 does not regulate the expression of the main proteins involved in this pathway (Fig. 6A). A critical step in the regulation of this pathway by mitogens is the phosphorylation of TSC2 by Akt. We further investigated whether E2F1 could activate TSC/mTORC1 signaling through activation of Akt. Previous reports have strongly indicated this possibility: E2F1 could induce Akt activity through transcription activation of the adaptor protein Grb2-associated binder [16]. However, phosphorylation of S6K induced by E2F1 was detected in conditions where Akt was not phosphorylated, suggesting that E2F1 does not regulate the TSC/mTORC1 pathway through activation of Akt (Fig. 6B). In agreement with this, phosphorylation of TSC2 at Thr1462, the target of Akt, was not detected at early stages of S6K activation. Inhibition of Akt expression with Akt1 siRNA did not change the effect of E2F1 on S6K phosphorylation, demonstrating that this kinase is not involved in this process (Fig. 6C).

In addition to Akt, Erk signaling could also regulate mTORC1 signaling by phosphorylation of the TSC1/TSC2 complex [13]. As E2F1 was shown to be able to activate Erk by up-regulating the expression of Ras guanine nucleotide exchange factors, we investigated whether Erk signaling was implicated in the growth response of E2F1 [22]. This possibility was discarded due to the fact that S6K activation was still detected in the presence of the specific MEK inhibitor PD98059 in the cells (Fig. 6D).

**Figure 7:**
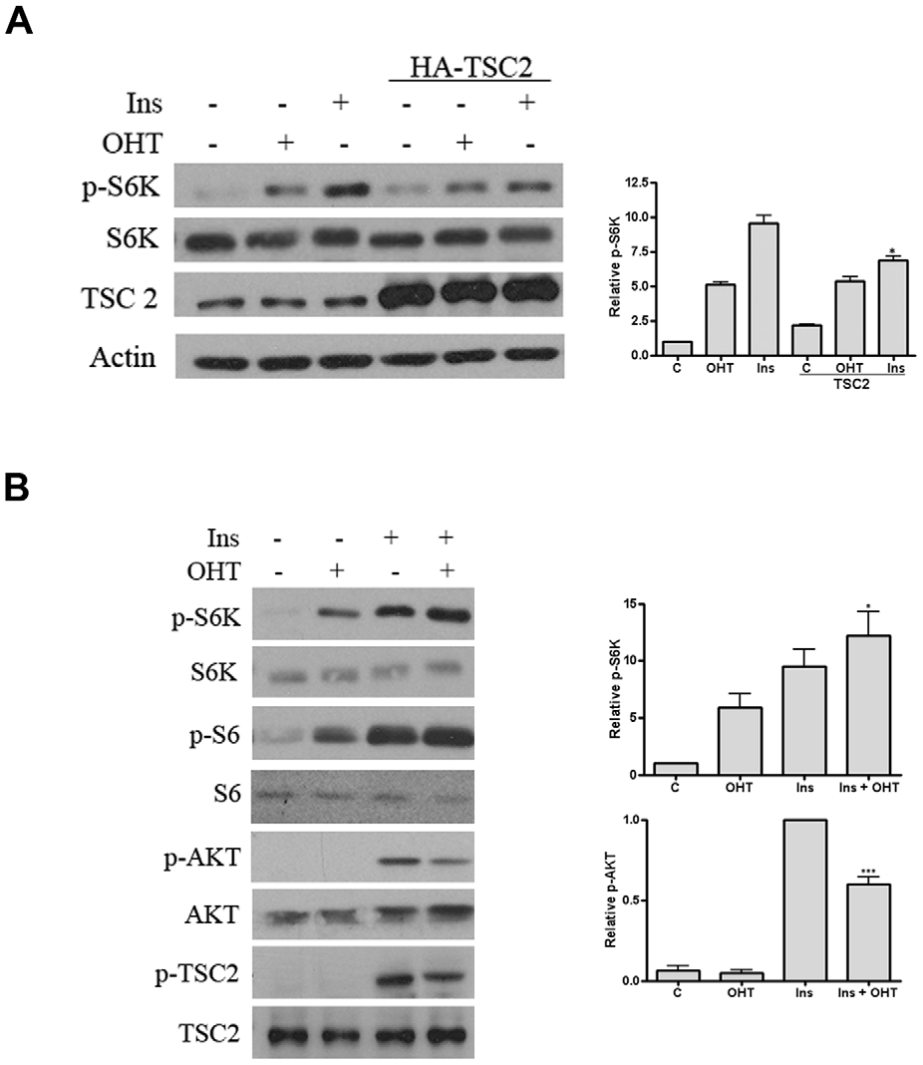
Effect of over-expression of TSC2 on the activation of S6K by E2F1. Expression of the indicated proteins was determined by Western blot analysis. (A) Stable ER-E2F1 U2OS cells were transient transfected or not with HA-TSC2 expression plamid, serum starved and treated with OHT (+) or not (−), and insulin (+) or not (−) for 6 h. Quantification of Western blot from four independent experiments is shown on the right panel. Statistically significant differences were obtained by comparison with no transfected HA-TSC2 cells. (B) Additive effects of E2F1 and insulin in mTOR signaling. (A) ER-E2F1 U2OS cells were serum-starved and treated with OHT (+) or not (−) for 4 hours, and (+) or not (−) with insulin (Ins) for 30 min. Quantification of Western blot from four independent experiments is shown on the right panels. *P<0.05 and ***P<0.001 Statistically significant differences were obtained by comparison with cells treated with insulin alone.

To answer the question whether TSC1/TSC2 complex is involved in the E2F1 growth response, we determined the effect of E2F1 on the S6K phosphorylation in the presence of over-expressed TSC2. As predicted from its negative role on Rheb activity, over-expression of TSC2 partially repressed the effect of insulin on the phosphorylation of S6K (Fig. 7A). However, over-expression of TSC2 did not interfere with the effect of E2F1. Indeed, E2F1 and insulin together had a stronger effect on the activation of S6K than alone, implying a complementary pathway for both signals (Fig. 7B).

### E2F1 induces the intercellular localization of mTORC1 to the late endosomes vesicles

**Figure 8:**
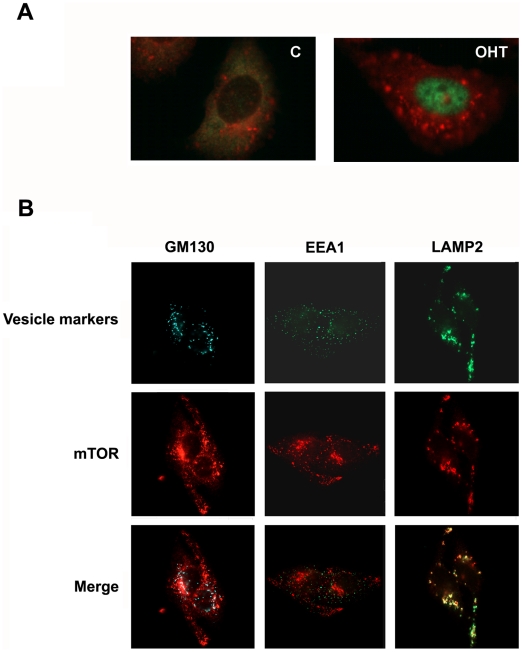
Effect of E2F1 on the mTOR localitation. (A) Stable ER-E2F1 U2OS cells were serum-starved and treated with OHT or not for 5 hours and immunostained for E2F1 (green) and mTOR (red). (B) Stable transfected ER-E2F1U2OS cells were serum-starved and treated with OHT for 5 hours and immunostained for indicated endogenous proteins.

**Figure 9:**
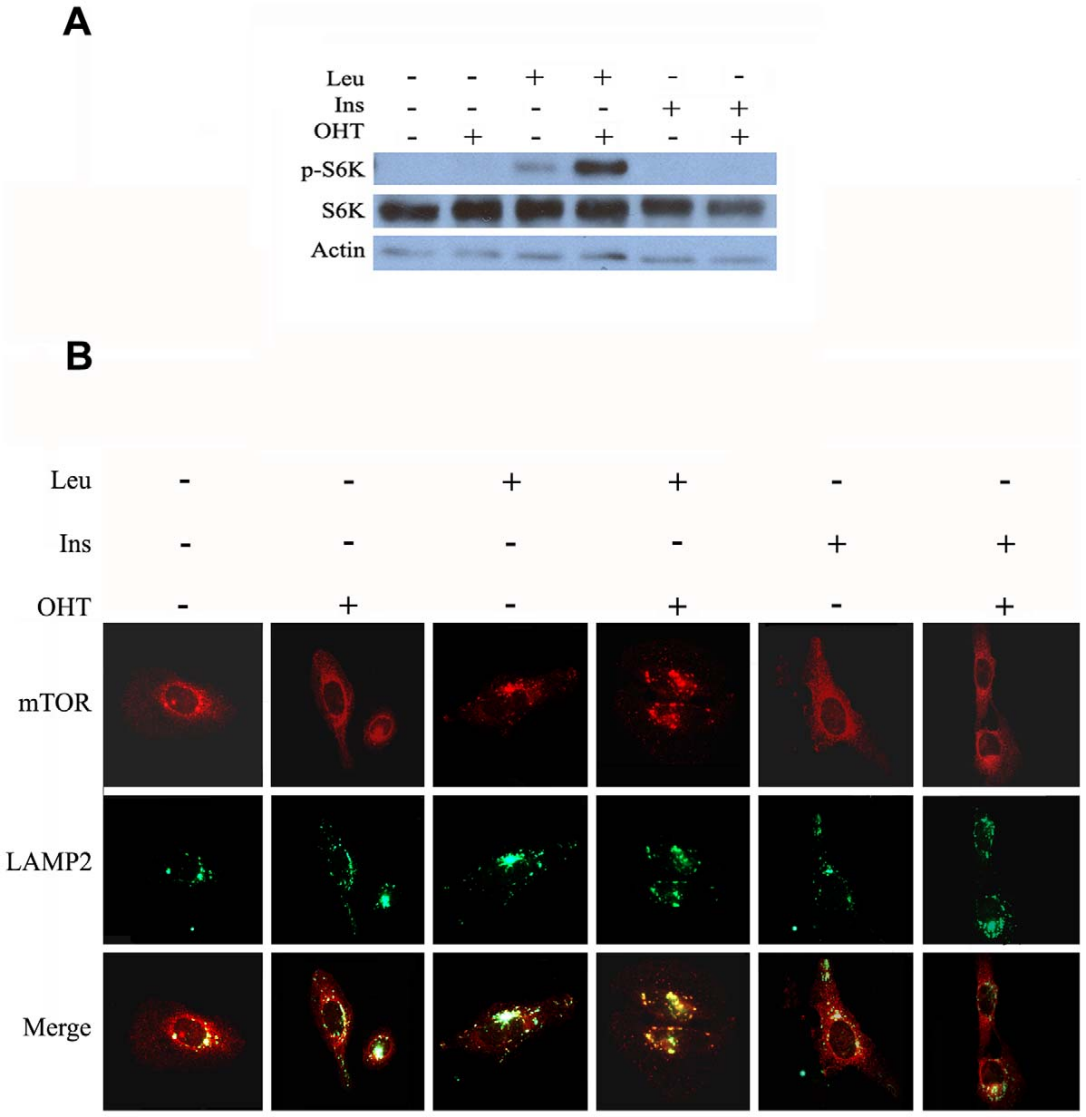
Effect of Leu on the activation and localitation of mTOR by E2F1. (A) Cells were serum and amino acids starved for 12 hours, and treated with OHT for 5 hours or/ and with insulin (Ins) or/and leucine (leu) for 30 min. Expression of the indicated proteins was determined by Western blot analysis. (B) Immunostained for mTOR (red) and LAMP2 (red) was achieved for experimetal conditions described in A.

Although TSC1/TSC2 complex is the main regulator of mTORC1 activity by mitogens, not all the pathways for mTORC1 activation go through this complex. It has been recently demonstrated that the Rag family of GTPases are essential mediators of mTORC1 activation in response to amino acids. As results of these investigations a model has been proposed that Rag GTPase regulate the intracellular localization of mTORC1. In this model, when amino acids are present, RagA and RagB recruit mTORC1 to the late endosome and lysosomal compartiments where Rheb is present and capable to interacte modulating mTORC1 activity [23]. To address whether E2F1 alters the mTOR1C1 targeting into vesicles, we investigated its subcellular localization under E2F1 induction. In E2F1 induced cells, more mTOR was found at the large vesicular structures comparing to non induced condition (Fig. 8A). Immunostaining studies using different subcellular markers demonstrated that mTORC1 is localized in enlarged vesicles that contain LAMP2, indicating its localization on the late endosomes. In contrast, when mTORC1 localization was compared with that of Golgi marker, GM130, or the early endosomal EEA1, colocalization was not observed (Fig. 8B). These data strongly suggest that E2F1 activates mTORC1 signaling by a mechanism that involves the translocation of mTORC1 to late endosomes. It is known that recruitment of mTORC1 to endosomes by the action of amino acid is an essential step for the activation of mTORC1 by mitogenic signals [24]. We further investigated whether the activation of mTORC1 by E2F1 requires the presence of amino acids. In the absence of amino acids, activation of E2F1 activity was not able to induce the phosphorylation of S6K neither alone nor in the presence of insulin (Fig. 9A). In contrast, high induction of S6K phosphorylation was observed when E2F1 was activated in the presence of leucine (Fig. 9A). In accordance with this result, translocation of mTORC1 to endosomes was only markedly detected in conditons where leucine was present, and futhermore the largest endosome vesicles were found when E2F1 activity was induced (Fig. 9B).

## Discussion

During cell proliferation, growth must occur to maintain homeostatic cell size. In this study, we showed for the first time that the mammalian E2F1, in addition to its role in proliferation and apoptosis, is also involved in cellular growth. The relationship between cell growth and E2F activity was investigated previously by others in *Drosophila*. In contrast to our results, in *Drosophila* the over-expression of dE2F accelerates the cell cycle without affecting growth [3]. The discrepancy between our results and those obtained using *Drosophila* could be due to the complexity of mammalian E2F systems. In *Drosophila*, the pRb pathway converges to the regulation of two members of the E2F family of transcription factors: an activator, dE2F1, and a repressor, dE2F2 [19]. However in mammals, the retinoblastoma signal pathway regulates the activity of eight different E2F members, with each of them playing a different role in cellular fate [2]. As a tentative model, it would be possible that, during the evolution process, E2F mammal members took on cellular functions that had previously involved proteins of the retinoblastoma pathway. According to this model, the growth function of Cyclin D evident in *Drosophila* would be partially replaced by E2F1 in mammals [4]. The growth activity of E2F1 was dependent on both E2F1's ability to bind DNA and to activate gene transcription. In accordance with this, a change in the transcription of E2F1-regulated genes has been reported in hypertrophy, a growth process that does not require cell division [6]. It is very likely that, as has been described for the proliferative and apoptotic functions, E2F1 is capable of inducing a gene expression program that could determine cellular growth.

Using a candidate protein approach, we investigated proteins involved in TSC/mTORC1 pathway, which levels of expression could differ after over-expression of E2F1. We showed that E2F1 did not regulate the expression of any of these clue proteins. The regulation of mTORC1 activity by mitogens requires Akt phosphorylation [11]. Interestingly the effect of E2F1 on the activation of mTORC1 did not depend on Akt phosphorylation. In fact, E2F1 and insulin together affected the activation of S6K stronger than alone, and not only that, they resulted in the reduction of Akt phosphorylation. The reduction could be due to the inhibitory effect of S6K on Insulin Receptor Susbstrate-1 [25]. We also ruled out any role of Erk2 on the activation of mTORC1 by E2F1. As Akt, Erk2 is capable of regulating the mTOR pathway by phosphorylation and inhibition of TSC2 complex, and previous studies have demonstrated that the activity of this kinase is regulated by E2F1 [13], [22].

A link between E2F1 and the mTORC1 pathway has been recently suggested in E2F1 transgenic mouse and human hepatocellular carcinomas (HCC) [26]. This study shows that dysplastic liver and hepatocellular carcinomas from transgenic mouse possess a higher mTORC1 activity together with a higher Akt phosphorylation than liver from wild type animals. Based on these results, and in contrast to the results showed here, the authors suggest an Akt-dependent activation of the mTORC1 cascade by over-expression of E2F1. Discrepancy on the role of Akt might be due to the fact that the samples analyzed in this study were obtained from neoplasic and pre-neoplasic tissues. Moreover, results from this study obtained by knock-down of the E2F1 protein suggest that E2F1 could also modulate Akt and mTORC1 expression. As pointed out above, we did not observed any change on the expression of these proteins in our experimental conditions. It is possible that regulation of Akt expression can occur at longer times after E2F1 induction; however, this will not be the responsible event for the activation of mTORC1 found at early moments of E2F1 activation.

In contrast to the effect of insulin on S6K phosphorylation, over-expression of TSC2 did not interfere with the action of E2F1. By interacting with Rheb, the TSC2 complex negatively regulates mTORC1 activity during mitogenic processes. In accordance with this role, previous studies demonstrate that over-expression of TSC2 or of the TSC1-TSC2 complex significantly reduces insulin-stimulated S6K phosphorylation [27]. The results presented in this study could suggest that the TSC2 complex does not participate in the signal transduction pathway activated by E2F1. As has been reported in several models, Rheb activity is reduced after the over-expression of TSC2 and essential for controlling mTORC1 activation; therefore, the lack of effect of TSC2 over-expression on E2F1 mTORC1 activation indicates that E2F1-induced signal pathway can compensate the inhibitory effect of TSC2 on Rheb [7], [27].

In order to elucidate the mechanism that E2F1 regulates mTORC1 activity, we investigated the intracellular localization of mTOR after E2F1 induction. Previous reports demonstrate that mTOR can be positively modulated by its translocation to the late endosome and lysosome, compartments where Rheb is present and capable of interacting with it [23]. This mechanism of mTORC1 regulation is used by amino acids to control cell growth [23], [24], [28]. Our results demonstrated that E2F1 is capable of inducing the translocation of mTORC1 to late endosome particles suggesting that this is the mechanism by which E2F1 regulates mTORC1 activation. How this process occurs is presently under investigation. One possibility is that transcriptional E2F1 targets could regulate, directly or indirectly, genes involved in the regulation of functions of the endosomal compartment, as per exemple those for the rag proteins. Interesting recent studies have shown that another transcription factor, p53, regulates endosomal formation by regulating the expression of TSAP6, Chmp4C, Caveolin-1 and DRAM [29], [30]. Because p53 is regulated by E2F1, it is possible that translocation of mTOR to late endosome was a p53 dependent process [2]. Moreover, E2F1 could modulate mTORC1 activity through the modulation of PLD. It has been demonstrated that this activity is required for Rheb activation of mTORC1 signaling to S6K. Activation of E2F1-targets genes could regulate PLD activity directly or by regulating proteins that interact with PLD1: cPKC, ARF and Rho [31]. Other possibilities should also be considered in future investigations of the mechanism by which E2F1 regulates mTORC1 activity, such as the involvement of other regulators of mTOR1 activity (PP2A, MAP4K3 or calcium) [32], [33].

Although E2F1 induced mTORC1 translocation to late endosomes, this process required the presence of amino acids. E2F1 alone was not able to replace amino acids in its function to induce mTORC1 translocation. However it enhanced the process. As a result of these experiments, we suggest two possible mechanisms of E2F1 action. E2F1 could be interacting with the amino acid signaling pathway and potenciating late endosomes formation or, E2F1 could be enlarging the endosome vesicles downstream of amino acid signaling.

From this study, E2F1 emerges as a key protein that integrates cell division and growth, which are essential for cell proliferation. We demonstrate for the first time that E2F1, in addition to its role in proliferation and apoptosis, is capable of regulating cellular growth. E2F1 regulates cell mass through modulation of mTOR activity by a mechanism involving the translocation of mTORC1 to the late endosomes. This exciting finding reveals the possibility that a new pathway could also mediate mTORC1 activation in mitotic conditions.

## Methods

### Cell Culture

U2OS and SAOS cell lines were cultured in DMEM media containing 10% fetal calf serum [34]. PC12 were cultured in DMEM media containing 6% fetal calf serum cells and 6% of horse serum. For ER-fusion proteins experiments, cells were serum-starved overnight, before 400 nM of 4-hydroxytamoxifen (Calbiochem, Germany) was added to activate E2F nucleus translocation. For assays using inhibitors, cells were pre-incubated for 1 h in serum-starved media in the presence of 5 µM of rapamycin (Sigma-Aldrich, USA) or 80 µM of PD98059 (Sigma-Aldrich, USA) prior the addition of 4-hydroxytamoxifen. As and when required, insulin (Sigma-Aldrich, USA) was added to the culture for 30 min at the final concentration of 200 ng/ml.

### Plasmid constructs and transfections

Plasmids pCMVHAER-E2F1, pCMVHA-E2F1 and pCMVHA-E2F3 were kindly provided by Dr. Kristian Helin [14]. pcDNAHATSC2 was kindly provided by Dr. George Thomas [35]. E2F1(1–284) and E132 plasmid were donated by Dr. Douglas Cress [20]. The pCMVHAER-E2F3 was obtained by removing the E2F1 coding region of pCMVHAER-E2F1 plasmid and replacing it with the E2F3, using the Bam H1 restriction site. pCMVCHAER was obtained by removing the E2F1 coding region and ligating the ends. pCMVHAER-(1–284) and pCMVHAER-E123 were obtained by PCR the E2F1 coding region of E2F1(1–284) and E132 plasmids and then introduced into the BamH1 site of the pCMVCHAER plasmid.

Plasmids were transfected with Lipofectamin (Invitrogen, USA) in accordance with the manufacture's instructions. To obtain the ER-E2F stable cell lines, cells were re-suspended and incubated in D-MEM selective medium, containing 10% serum and 750 µg/ml Geneticin Selective Antibiotic (G418) (Gibco, UK). After 15 days, the transfected stable cell line was obtained and maintained in the D-MEM selective medium). 150 nM of E2F1 siRNA (Santacruz Biotechnology, USA) or non-silencing siRNA (NS siRNA) (Santacruz Biotechnology, USA) was transfected using Lipofectamin, Akt1 siRNA (Ambion, USA) was transfected using the TransIT-siQUES (Mirus, USA) following manufacture's instructions. Luciferase activity was measured using the Luciferase Assay kit in accordance with the manufacturer's recommendations (Promega, USA).

### Western blotting

Cells were washed and harvested with PBS. Cell pellets were re-suspended and incubated for 30 min at 4°C in lysis buffer (20 mM Tris-HCl pH 8.0, 10 mM EDTA, 2.5 mM MgCl_2_, 1% Triton X100 with a supplement 1/100 dilution of phosphatase and protease inhibitor cocktail (Sigma-Aldrich, USA). Equal amounts of protein lysate were subjected to 10% sodium dodecylsulphate polyacrylamide gel electrophoresis and electrophoretically transferred to PVDF membranes. The membranes were then blocked with 5% non-fat milk and 5% BSA solution in PBS-T (PBS with 0.1% Tween 20) for 1 hour at room temperature and immunoblotted for 2–12 hours (information about antibodies is on supplementary information). Primary antibody and secondary horseradish peroxidase-conjugated antibody incubations were performed in 5% non-fat milk and 5% BSA solution in PBS-T. Blots were developed using an Enhanced Chemiluminescence kit (Amersham, UK).

### Flow cytometry

Cells were serum-starved for 24 hours and then treated as described in the figure legend. At the indicated time, cells were collected by trypsinization, washed, re-suspended in cold PBS, and fixed by addition of ethanol to a final concentration of 70%. Fixed cells were re-suspended in PBS containing 0.1% Triton, 0.1% serum and 0.1 mg/ml RNAse (Roche, USA), DNA was labeled with 0.05 mg/ml of propidium iodide (Sigma-Aldrich, USA) and incubated for 30 min at 37°C in a dark place. Cells were analyzed using a flow cytometry (FACScan, Beckton Dickinson) using WinMDI 2.8 software.

### Immunofluorescence analysis

Cells were plated onto glass coverslips and grown under the indicated conditions. Then, cells were fixed in 4% paraformaldehyde for 30 minutes at 4°C, permeabilized with 0,3% Triton X-100 (Sigma-Aldrich, USA) and glycine 20 mM in phosphate-buffered saline (PBS) for 10 minutes at room temperature, and incubated in blocking solution (1% bovine serum albumin in PBS glycine 20 mM) for 15 minutes at room temperature. As indicated, mTOR rabbit monoclonal IgG (Cell Signaling Technology, USA) diluted in blocking solution at 1∶150, or E2F1 mouse monoclonal IgG (Santa Cruz Biotechnology, USA) diluted at 1∶400 or EEA1 mouse IgG, mouse anti-GM130 (BD Biosciences Pharmingen, USA) or mouse anti-LAMP2 (BD Biosciences Pharmingen, USA) diluted at 1∶300 were incubated for 60 minutes at 37°C. As a secondary antibody, we used fluorophore-labeled Rhodamine Red goat anti-rabbit IgG and Alexa Fluor 488 goat anti-mouse IgG (Molecular Probes, USA), incubated at 1∶300 dilution in blocking solution for 45 minutes at 37°C. For nuclei staining, cells were then washed for 10 minutes at room temperature in PBS with 1 µg/ml Hoechst 33258 (Molecular Probes). Coverslips were washed and mounted in Prolong Gold antifade reagent (Molecular Probes), and cells visualized under fluorescent microscopy. mTORC1 localization was observed at an excitation/emission wavelength of 560/580 nm, cell nuclei at 350/460 nm, and the others proteins marked with the Alexa fluor probe were observed at 500/520 nm.

### Statistic analysis

Data was analysed by GraphPad Prism4 software. Results are presented as Mean±S.E.M for n = 4. Experimental data sets were compared by a two-sampled, two-tailed and unequal S.D. Student *t* Test. Values of *P<0.05, **P<0.01 and ***P<0.001 were considered statistically significant.

## Supporting Information

Figure S1 **Effect of rapamycin on the expression levels and translocation of ER-E2F1 to the nucleus.** Stable ER-E2F1 U2OS cells were serum-starved and treated with OHT (+) or not (−) for 6 hours (OHT) in the presence or in the absence of rapamycin (rap). (A) Expression of the indicated proteins was determined by Western blot analysis. (B) Cells were immunostained for E2F1 protein (green) and Hoechst stain (blue).
(DOC)

Figure S2 **Effect of tamoxifen on E2F3 transcriptional activity.** U2OS cells were transient transfected with ER-E2F3 and [E2F]_3_-Luc vectors. After serum-starved, cells were treated with OHT (+) or not (−) for 12 h and luciferase activity was measured for both conditions. Statistically significant differences were obtained by comparison with untreated cells.
(DOC)
