# Supplementary figures and images for: E2F1 Regulates Cellular Growth by mTORC1 Signaling
Source: PLoS One. 2011 Jan 24;6(1):e16163. doi: 10.1371/journal.pone.0016163 (PMC3026008; doi:10.1371/journal.pone.0016163)

##

**Figure S1.**


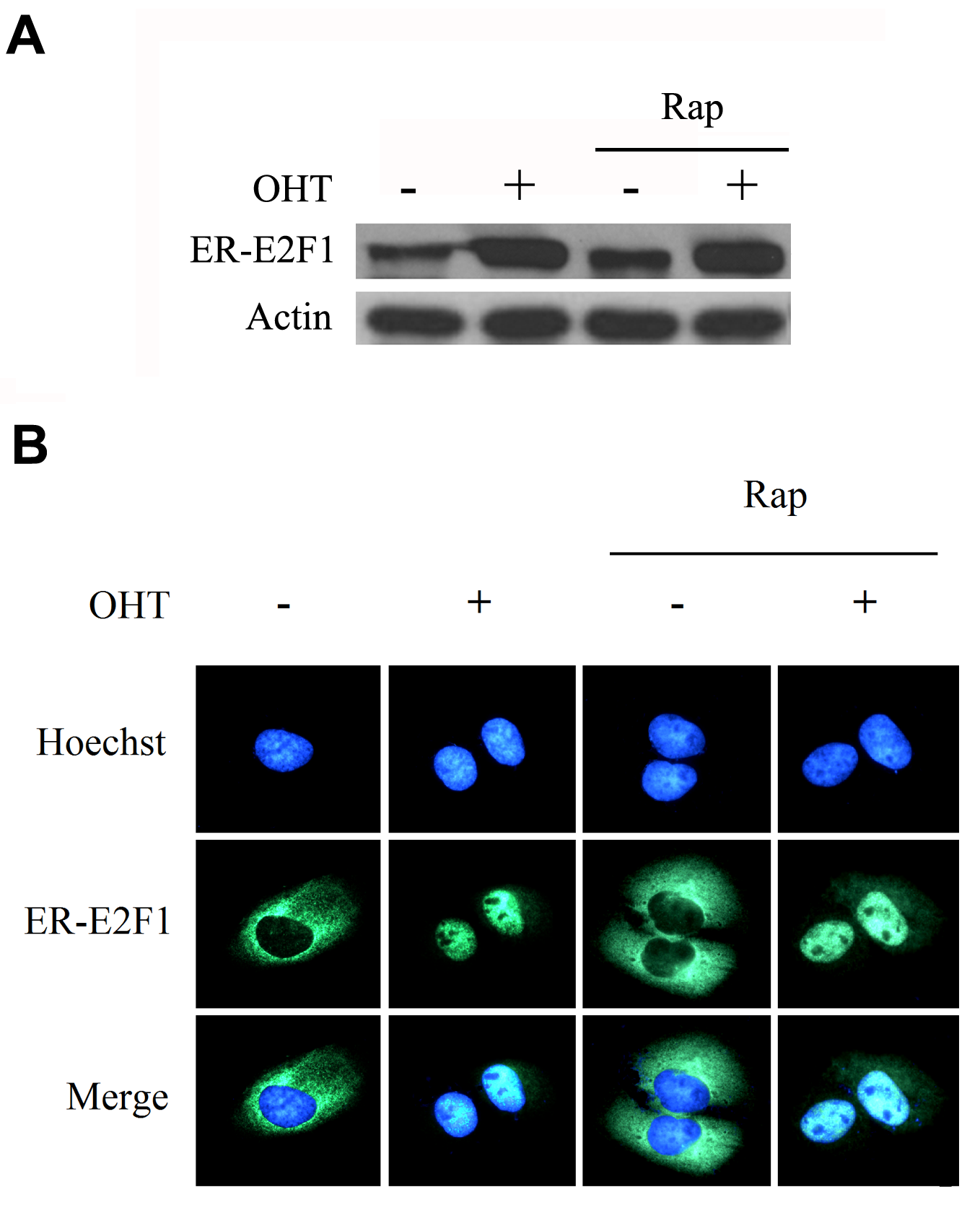

Supplement: Figure S1 — Effect of rapamycin on the expression levels and translocation of ER-E2F1 to the nucleus. Stable ER-E2F1 U2OS cells were serum-starved and treated with OHT (+) or not (−) for 6 hours (OHT) in the presence or in the absence of rapamycin (rap). (A) Expression of the indicated proteins was determined by Western blot analysis. (B) Cells were immunostained for E2F1 protein (green) and Hoechst stain (blue). (DOC) [file pone.0016163.s001.doc]

**Figure S2.**


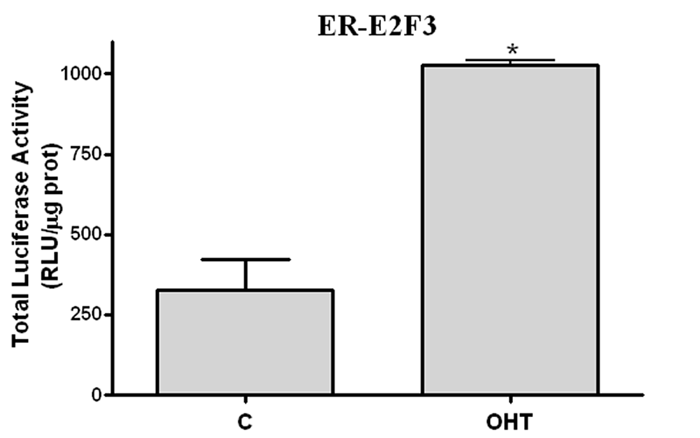

Supplement: Figure S2 — Effect of tamoxifen on E2F3 transcriptional activity. U2OS cells were transient transfected with ER-E2F3 and [E2F]3-Luc vectors. After serum-starved, cells were treated with OHT (+) or not (−) for 12 h and luciferase activity was measured for both conditions. Statistically significant differences were obtained by comparison with untreated cells. (DOC) [file pone.0016163.s002.doc]
